# Case Report: Premature lactation in Jersey heifers after intercontinental air transport

**DOI:** 10.3389/fvets.2025.1601524

**Published:** 2025-05-22

**Authors:** Guanglei Liu, Eryl Done, Joyce Ip, Cheuk Ming Li, Kate Flay, Ákos Kenéz

**Affiliations:** ^1^Jockey Club College of Veterinary Medicine and Life Sciences, City University of Hong Kong, Hong Kong SAR, China; ^2^CityU Farm, Jockey Club College of Veterinary Medicine and Life Sciences, City University of Hong Kong, Hong Kong SAR, China; ^3^Department of Infectious Diseases and Public Health, City University of Hong Kong, Hong Kong SAR, China; ^4^Department of Veterinary Clinical Sciences, City University of Hong Kong, Hong Kong SAR, China

**Keywords:** cattle, stress, udder edema, mycotoxin, long-distance transport

## Abstract

The establishment of a new teaching and research dairy farm at City University of Hong Kong (Hong Kong SAR, China) necessitated the importation of pregnant dairy heifers from Australia. On 20 September 2022, a cohort of 24 pregnant heifers arrived by air to CityU Farm. Commencing shortly after arrival, during the subsequent month all heifers exhibited abnormal udder development resembling cows within 2 weeks pre-parturition, despite being 10–17 weeks from calving. Further clinical examination showed excessive teat edema, ventral abdominal edema and milk leakage. Additionally, serum biochemical analysis identified elevated cortisol and prolactin levels, accompanied by reduced cholesterol and triglyceride levels. These observations indicated that premature lactation in the heifers could be associated with transport-induced stress, hormonal imbalances, and potential zearalenone contamination in the feed. Interventions were implemented upon the appearance of clinical signs, including the reduction of artificial lighting in the barns to reduce solar-induced stress, removal of concentrate from the diet with only timothy hay retained, and implementing timely monitoring and treatment of mastitis cases. The majority of the heifers calved successfully as expected, with the exception of one premature calving case (approximately 6 weeks early). Notably, half of the heifers that experienced premature lactation exhibited reduced colostrum quality at calving post-recovery with 50% (of 20 sampled) having a %Brix value of <22, (mean %Brix value of 22.13 ± 4.20). The findings emphasize the physiological challenges associated with international cattle transport and underscore the need for research-driven strategies to improve livestock acclimatization, welfare, and management during and after transportation.

## 1 Introduction

**Figure 1:**
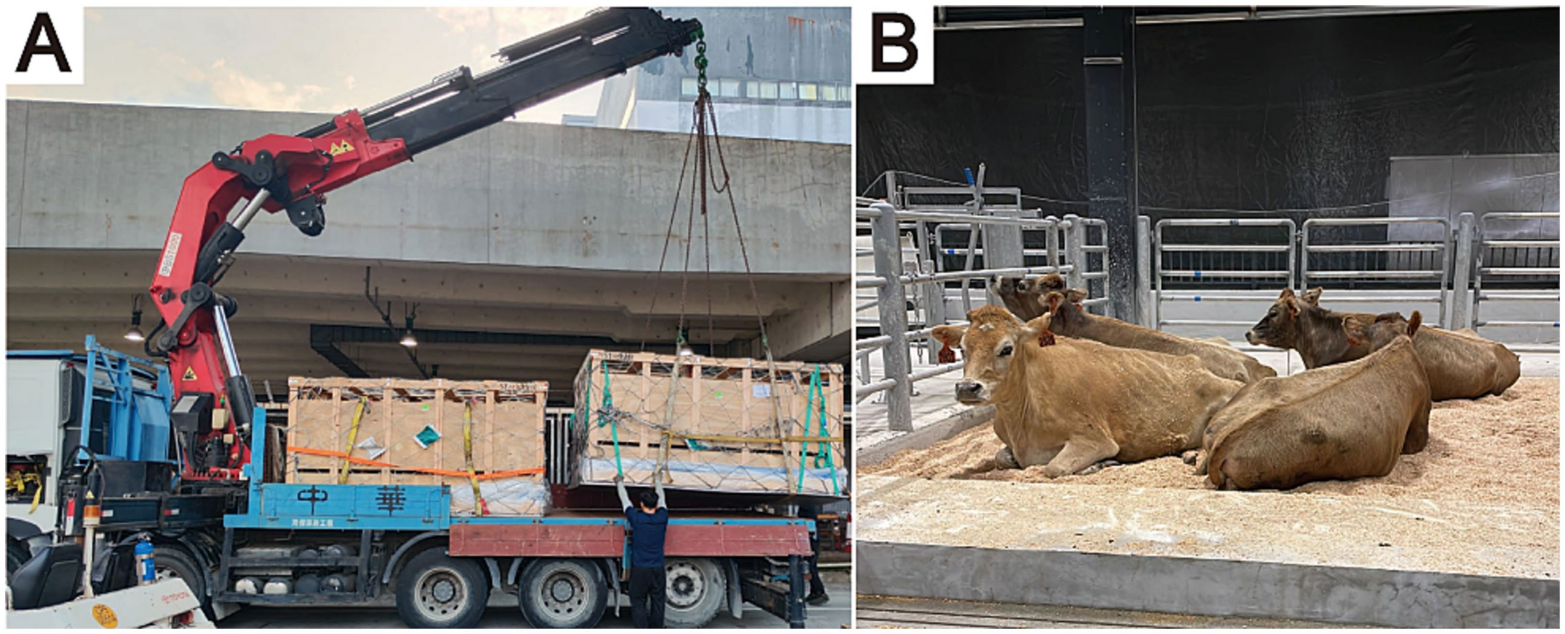
Arrival and housing of Jersey heifers. **(A)** Jersey heifers unloading at Hong Kong International Airport in transport crates. **(B)** The first batch of Jersey heifers at the City University of Hong Kong (CityU) Dairy Farm.

Veterinary and animal science universities commonly own a model dairy farm where dairy cows play a vital role in supporting teaching, research, and the production of high-quality dairy products (1). To enhance its internationally accredited veterinary education program, City University of Hong Kong (CityUHK) established a dairy farm in 2022, importing Jersey heifers from Australia. This initiative addressed the challenges of limited ruminant accessibility in Hong Kong’s urban environment (2), providing essential resources for teaching, research, and small-scale dairy production, including premium products like milk and ice cream sold on campus. The importation of these cattle went as planned (Figure 1), however, shortly after arrival, they exhibited unexpected health issues, notably udder edema and premature lactation. Premature lactation, characterized by early udder development and milk production before calving, has been linked to long-distance transport stress, hormonal imbalances, and dietary changes (3), while the underlying mechanisms remain poorly understood. To our knowledge, this is the first documented case of premature lactation in Jersey heifers after intercontinental air transport.

This case study investigates the causes of premature lactation in imported Jersey heifers, focusing on transport-induced stress, hormonal disruptions, and potential feed-related factors. By proposing management strategies to improve cattle welfare and health performance, the findings aim to contribute to best practices for livestock importation, particularly in the context of increasing global trade and the growing prevalence of intercontinental animal transport. The study underscores the need for research-driven approaches to mitigate health challenges and ensure the well-being of dairy cattle in academic and teaching settings.

## 2 Case description

### 2.1 Patient information

On 20 September 2022, a cohort of 24 pregnant Jersey heifers (aged 22–30 months, gestational age: 220–250 days) were imported from Victoria, Australia to CityUHK Dairy Farm by air. The estimated transit time to the farm was approximately 16 h; however, due to delays in both the flight and local transportation, the actual transit time extended to 20 h. Upon arrival, the heifers were housed under the supervision of an experienced herd manager (who is also a registered veterinarian) and subjected to close monitoring to assess health and productivity parameters, as detailed in Table 1.

**Table 1:** Timeline of the case.

Timepoint	Event
Before flying	All heifers were confirmed healthy, free from infectious diseases and parasitic infestations, following a five-month isolation period and an additional one-month pre-embarkation quarantine and testing before official export via an international flight
In transit	The heifers were allocated into four crates, with six heifers per crate (Figure 1A). The entire journey lasted approximately 20 h, including 10 h of actual flight time and 10 h of ground transportation to and from the airport
Arrival at the farm (day 1 & day 2)	No abnormal behavior indicative of stress was observed in the heifers. Subsequently, they were provided with clean water *ad libitum* under ambient temperatures ranging from 27 to 32°C. During the first two days, all heifers were provided *ad libitum* of hay, with no concentrate supplementation
Day 3—clinical signs began to appear	The diet of all heifers was transitioned from hay to a combination of concentrate (corn, soybean meal, soya hulls and mineral premix) and hay
Day 7	Teat edema was initially observed in 7/24 heifers, with one case accompanied by milk leakage
After the onset of clinical signs	Artificial lights in the barns were switched off to minimize light exposure, and concentrate was removed from the daily diet upon the appearance of clinical signs, only hay provided thereafter
A month after arrival	All 24 heifers developed varying clinical signs within one month, including (1) teat edema, (2) ventral edema, and (3) running milk. Additionally, three heifers developed mastitis subsequent to running milk
2–3 months after arrival	Gradual recovery was observed, with udder edema and milk letdown subsiding in most heifers
After calving	Most of the heifers calved as expected excluding one which had a premature calving (6 weeks early). However, the colostrum quality of 50% (*n* = 10) of sampled heifers (*n* = 20) was poor with a %Brix < 22 (mean %Brix = 22.13 ± 4.20). A further two did not produce any colostrum to sample due to premature calving (*n* = 1) and mastitis (*n* = 1). At calving, several of the heifers exhibited very poor udder conformation with seven out of 24 (29%) having udder depth level of below the point of the hock and five out of 24 (21%) having broken udder support (cleft of median suspensory ligament poorly or not visible) (Figure 2D)
Calving—1st lactation	The average first lactation milk yield of the heifers was 7,229 kg in 305 days, with high-quality milk composition in homogenized milk (fat: 5.0%, protein: 3.8%, calcium: 130 mg/mL)

### 2.2 Clinical findings

**Figure 2:**
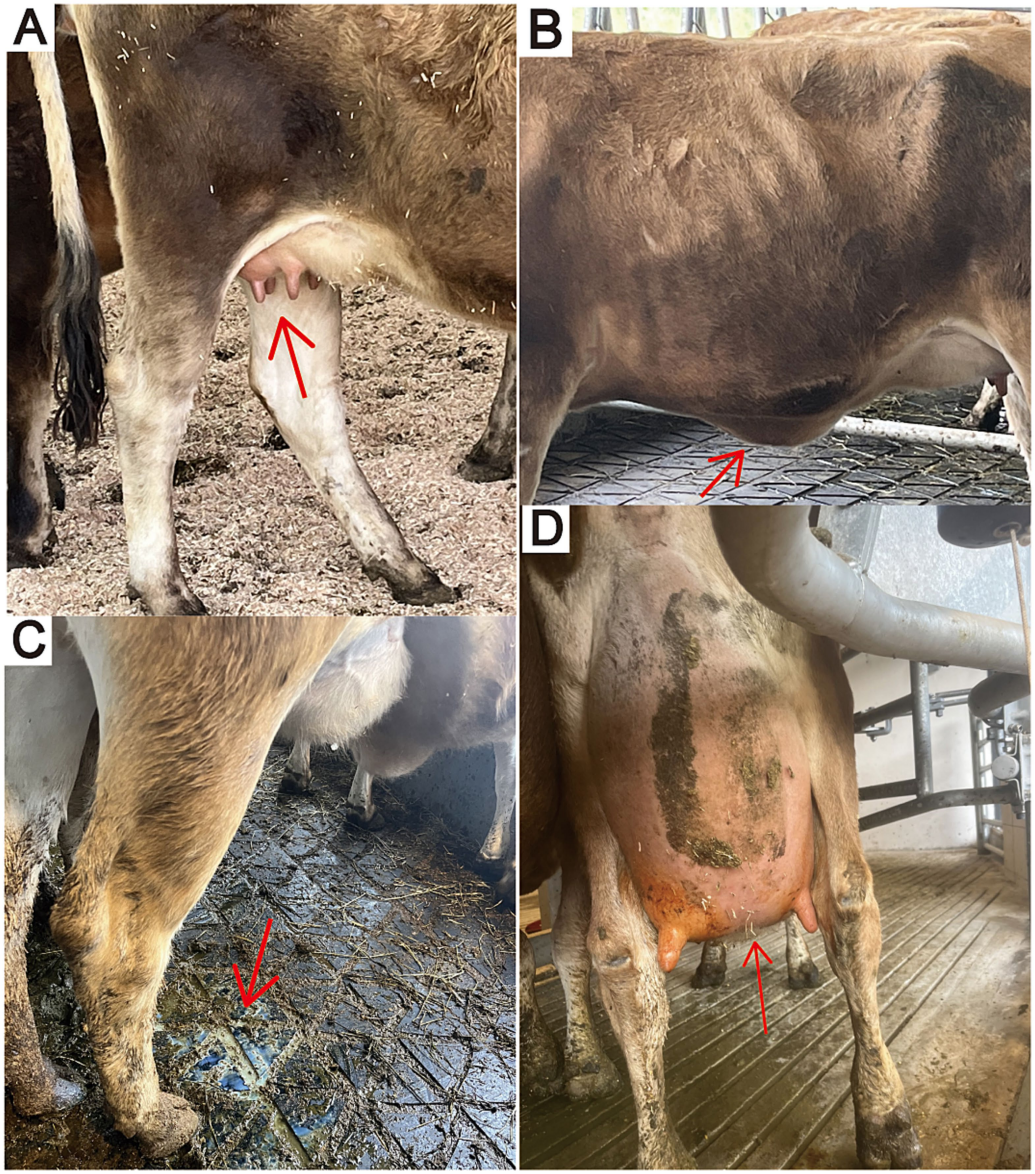
Clinical signs of premature lactation in Jersey heifers. **(A)** Teat edema. **(B)** Ventral edema. **(C)** Running milk. **(D)** Poor udder conformation (weak udder support and udder floor below hock level) after subsequent calving.

Following a thorough examination by two registered veterinarians, all 24 heifers gradually developed varying degrees of clinical manifestations, including teat edema (Figure 2A), ventral edema (Figure 2B), and running milk (Figure 2C) within the first month after arrival (see timeline, Table 1). We also recorded the weather conditions at CityUHK Farm on the day of arrival, with an average temperature of 31.2°C and 73.3% relative humidity, resulting in severe heat stress (4) with a temperature-humidity index (THI) of 84.6. These conditions were markedly hotter and more humid than those in Victoria, Australia, where the animals had previously experienced winter conditions (September 2022 monthly average: 11°C, 79% humidity, THI = 53).

Due to the limited case studies on premature lactation in heifers following long-distance air transport, we hypothesize that several factors may have contributed, including transport-induced and heat stress from intercontinental travel leading to metabolic disruptions and immune suppression, circadian rhythm alterations caused by changes in photoperiod, and environmental factors such as Hong Kong’s high-temperature and high-humidity climate, which may have induced heat stress and increased the risk of feed contamination with mycotoxins. To further investigate, whole blood hematological parameters and serum biochemistry profiles were analyzed to assess the heifers’ overall health status. Serum cortisol and prolactin concentrations were measured to evaluate stress levels, and mycotoxin analysis of the hay was conducted to identify whether potential feed contamination existed.

### 2.3 Diagnostic assessment

#### 2.3.1 Blood parameters

Blood samples were collected 8 days post-arrival (30 September 2022) using both EDTA and serum vacutainer tubes (BD Vacutainer, Franklin Lakes, NJ, United States). All samples were transported to the City University of Hong Kong’s Veterinary Diagnostic Laboratory within 2 h for subsequent hematological and biochemical analysis, with the rest stored at −20°C until further analysis. Blood samples with EDTA were inverted several times to ensure homogeneity. Hematological analysis was performed using an automated hematology analyzer (Sysmex XT-2000i, Japan), while serum biochemical analysis was conducted using a biochemical analyzer (Cobas c311, Roche Diagnostics, Switzerland). Cortisol and prolactin levels were measured using bovine-specific ELISA kits (FineTest, China) following the manufacturer’s instructions and the methodology described by Di Meo et al. (5).

Hematological and serum biochemistry analyses revealed significant abnormalities in these prematurely lactating heifers (Table 2). Key findings included abnormal red blood cell parameters (RBC, MCV, MCHC), elevated neutrophil counts (50% of heifers), and WBC abnormalities (45.83% of cases). Serum analyses showed severely reduced cholesterol, low triglycerides, and decreased globulin levels, indicating impaired energy metabolism and immune function. Cortisol and prolactin levels were markedly elevated, with serum concentrations of cortisol (66.8 ± 63.1 ng/mL) and prolactin (272.2 ± 87.4 ng/mL) exceeding the reported normal ranges for primiparous dairy cows (cortisol: 4.61–10.29 ng/mL; prolactin: 1.43–2.46 ng/mL) described by Di Meo et al. (5). Notably, cortisol levels exhibited high variability, ranging from 10.37 to 269.05 ng/mL. These results highlight profound physiological and metabolic disruptions linked to premature lactation, further supporting stress and hormonal imbalance as key contributors to clinical symptoms.

**Table 2:** Overall blood metabolic status in premature lactation heifers.

Parameters	Mean ± SD^a^	PAV^b^, %	Reference range^n^
Hematological parameters			
RBC^c^, ×10^12^/L	6.54 ± 0.89	100	13.6–23.70
Hemoglobin (Hb), g/L	110.92 ± 14.29	12.5	87.0–124.0
Hematocrit (HCT), %	30.77 ± 4.10	8.33	28.0–41.0
MCV^d^, fL	47.17 ± 2.60	100	16.0–22.0
MCH^e^, pg	17.00 ± 0.94	100	5.0–7.0
MCHC^f^, g/L	360.50 ± 7.28	100	320.0–340.0
Parasites	0.00 ± 0.00	0	n.a
WBC^g^, ×10^9^/L	6.92 ± 1.92	45.83	7.20–17.7
Neutrophils, ×10^9^/L	2.00 ± 1.23	50	1.90–9.50
Band neutrophils, ×10^9^/L	0.00 ± 0.00	0	0.00–0.10
Lymphocytes, ×10^9^/L	4.33 ± 1.5	12.5	2.60–11.70
Monocytes, ×10^9^/L	0.18 ± 0.14	0	0.00–0.90
Eosinophils, ×10^9^/L	0.35 ± 0.42	8.33	0.00–0.80
Basophils, ×10^9^/L	0.05 ± 0.06	0	n.a
Platelet count, ×10^9^/L	435.79 ± 300.6	29.17	247–912
PCV^h^, %	30.71 ± 4.21	8.33	24–37
TPP^i^, g/L	69.46 ± 3.02	0	n.a
Serum biochemistry parameters			
Calcium, mmol/L	2.53 ± 0.10	0	2.23–2.73
Phosphorus, mmol/L	1.90 ± 0.15	8.33	1.49–2.36
Bicarbonate, mmol/L	23.66 ± 4.64	33.33	24–32
Urea, mmol/L	3.74 ± 1.24	25	2.5–6.4
Creatinine, mmol/L	67.83 ± 15.29	25	44.2–79.6
Cholesterol, mmol/L	2.05 ± 0.31	100	3.3–8.6
Triglyceride, mmol/L	0.30 ± 0.06	79.17	0.1–0.2
Glucose, mmol/L	3.44 ± 0.45	16.67	2.9–4.2
Total protein, g/L	67.33 ± 4.94	33.33	69–86
Albumin, g/L	38.67 ± 2.73	12.5	31–41
Globulin, g/L	28.67 ± 3.47	66.67	31–54
Total bilirubin, μmol/L	1.73 ± 0.53	54.17	1.7–3.4
ALP^j^, U/L	102.99 ± 44.69	33.33	29–111
GGT^k^, U/L	14.20 ± 4.35	0	9–50
AST^l^, U/L	65.30 ± 15.23	37.5	61–162
CK^m^, U/L	90.31 ± 15.52	15	76–376
Serum cortisol and prolactin concentration			
Cortisol, ng/mL	66.8 ± 63.1	100	4.61–10.29
Prolactin, ng/mL	272.2 ± 87.4	100	1.43–2.46

a Mean ± SD: data are presented as the mean value ± standard deviation.

b PAV, %, percentage of values outside the recommended range, calculated as the number of abnormal values divided by total observations, multiplied by 100.

c RBC, red blood cell count.

d MCV, mean corpuscular volume.

e MCH, mean corpuscular hemoglobin.

f MCHC, mean corpuscular hemoglobin concentration.

g WBC, white blood cell count.

h PCV, packed cell volume.

i TPP, total plasma protein.

j ALP, alkaline phosphatase.

k GGT, gamma-glutamyl transferase.

l AST, aspartate aminotransferase.

m CK, creatine kinase.

n Reference values of the Veterinary Diagnostics Laboratory of City University of Hong Kong; n.a, not detected.

#### 2.3.2 Feed contamination

Timothy hay samples were collected on 06 October 2022 and submitted to SGS Hong Kong Limited (Hong Kong, China) for mycotoxin contamination analysis. These were analyzed for aflatoxin B1, zearalenone, ochratoxin A, deoxynivalenol, T-2 toxin, HT-2 toxin, fumonisin B1, and fumonisin B2 following guidelines by Anfossi et al. (6).

Two out of the three hay samples tested positive for zearalenone (ZEN) (0.129 and 0.051 mg/kg, respectively), suggesting this batch of timothy hay was contaminated with ZEN. The rest of the mycotoxins were negative in the hay samples.

### 2.4 Therapeutic intervention

To address premature lactation, all affected heifers were managed without artificial lighting in the barns so that they were only subjected to normal daylight hours and did not have artificially extended light exposure. Concurrently, dietary adjustments were implemented to mitigate metabolic stress. Initially, the heifers were provided *ad libitum* timothy hay and water for the first 2 days post-arrival. Subsequently, their diet was transitioned to include a concentrate (consisting of corn, soybean meal, soya hulls, and mineral premix). Following the onset of clinical signs, the concentrate was withdrawn to reduce protein, energy and carbohydrate intake, and only timothy hay was provided thereafter. The hay was fed continuously until mycotoxin test results were available, after which it was replaced with a fresh, uncontaminated batch. A daily udder management routine was adopted including udder disinfection with an iodine-based post-dip spray (1.0% titratable iodine and 10% multi-emollient glycerol) twice daily to reduce the risk of mastitis caused by milk leakage.

Clinical mastitis was diagnosed based upon abnormal udder inflammation, causing uneven quarters, and milk of abnormal colour and consistency upon stripping out the affected quarters, followed by the detection of specific mastitis pathogens through microbiological examination. Among the 24 heifers in premature lactation, three (Nos. 12, 13, and 18) were diagnosed with clinical mastitis between 5 to 12 weeks post-import (around 3 months antepartum). Subsequent microbiological analysis revealed infections predominantly caused by *Streptococcus uberis*, *Actinomyces* spp., and *Corynebacterium* spp.

Mastitis treatment for the affected heifers (Nos. 12, 13, and 18) involved administering Syntocin^™^ (oxytocin, 10 IU/mL) as 5 mL intramuscular doses every 8–12 h and stripping out the affected quarters followed by intramammary infusion of Maxalac LC^™^ (cefuroxime sodium, 92.2 g/kg suspension) twice daily for 5–7 days. For two heifers (Nos. 13 and 18) this was combined with systemic administration of Mamyzin^™^ (Penethamate Hydriodide 269.5 mg/mL powder and solvent for suspension) via intramuscular injection once daily for 3–5 days. One heifer (No. 12) was treated with the systemic administration of Tribactral S^™^ (sulfadiazine-trimethoprim, 40 mg/mL) by intravenous injection every 24 h. Metacam^™^ (meloxicam, 0.5 mg/kg) was administered as a single intravenous injection to all three heifers at the onset of clinical signs. At the cessation of clinical signs of mastitis all three affected heifers were dried off with intramammary infusion of Cepravin^™^ (cefalonium, 250 mg/3 g) administered to all quarters, followed by Orbeseal^™^ (bismuth subnitrate, 2.6 g) teat sealant application to prevent new infections.

### 2.5 Follow-up and outcomes

The overall therapeutic methods appeared to be effective. Although nearly all the heifers recovered after the intervention, one case of premature calving occurred (6 weeks early), and their colostrum quality was highly affected with 50% (*n* = 10) of sampled heifers (*n* = 20) with a %Brix < 22 (mean %Brix = 22.13 ± 4.20) (7). A further two did not produce any colostrum to sample due premature calving (*n* = 1) and mastitis (*n* = 1). At their subsequent calving following recovery the udder conformation of the heifers was very poor for primiparous heifers with seven having udder depth level of below the point of the hock and five having broken udder support (cleft of median suspensory ligament poorly or not visible) (Figure 2D). Of the three heifers that had had clinical mastitis prepartum during premature lactation, one heifer (No. 12) had a non-functional back right quarter after calving and one (No. 13) had a light back left quarter. Their other udder quarters remained normal. The third heifer (No. 18) recovered fully and had no udder abnormalities post calving. Surprisingly, all recovered heifers exhibited excellent milking performance. Follow-up observations of daily milk production in their first parity indicated favorable post-calving outcomes. Milk composition analysis of homogenised milk (pasteurized and packaged by Trappist Dairy Limited, Hong Kong) indicated high nutritional quality (fat: 5%, protein: 3.8%, calcium: 130 mg/mL), with an average yield of 7,229 liters per cow in 305 days. The raw milk produced met the standards set out in the Hong Kong legislation for pasteurization and human consumption. These findings suggest that, despite initial stress and health challenges, the heifers successfully acclimated to their new environment and achieved remarkable lactation performance. However, the very poor udder conformation of some heifers is likely to lead to premature culling and therefore reduced lifetime yield.

## 3 Discussion

Currently, most studies on premature lactation in dairy heifers have focused on cases associated with prolonged sea transport (3). However, multiple factors can contribute to premature lactation in heifers, including hormonal imbalances, stress-induced metabolic disruptions, and dietary changes. In this case, long-distance intercontinental air transport likely induced serious stress, leading to elevated cortisol and prolactin levels, which disrupted normal mammary gland development, ultimately causing premature lactation. Additionally, environmental factors such as the high-temperature and high-humidity climate of Hong Kong may have increased the risk of heat stress (8) and feed contamination with mycotoxins (9, 10). Zearalenone, which is also known as estrogenic mycotoxin, with specific functions of mimicking estradiol and binding to estrogen receptors, potentially accelerated abnormal mammary development and premature lactation. Although low doses of ZEN have minimal impact on the growth performance of adult dairy cows due to their rapid excretion through urine and milk, the effects on pregnant heifers appear to be more significant (11). After long-distance intercontinental air transport, cattle are prone to stress-induced metabolic disorders and immune suppression (12), which, in turn, exacerbated the detrimental effects of ZEN on the heifers, ultimately contributing to the development of udder edema. Breed susceptibility may represent an additional contributing factor. Further research is needed to verify whether Jersey heifers are more predisposed to develop premature lactation compared to other breeds.

The laboratory data indicated disturbances in lipid metabolism. Cholesterol acts as a key substrate for the synthesis of vital components like lipoproteins and steroid hormones (13). In this study, all 24 heifers had low plasma cholesterol levels, indicating slight cholesterol homeostasis disruption. Triglycerides (TG), as a crucial energy source (14), are derived from the re-esterification of free fatty acids by the liver under normal physiological conditions. However, our findings indicated that approximately 75% of the heifers exhibited low TG concentrations, which was linked to malnutrition and edema (15) and can also be explained by decreased metabolic capacities of the liver (16). Elevated activities of hepatic enzymes, such as aspartate aminotransferase (AST) and alkaline phosphatase (ALP), were observed in some heifers, reflecting varying degrees of liver dysfunction. Further, hepatic impairment could also hinder lipid oxidation, potentially leading to the accumulation of non-esterified fatty acids (NEFA) and ketone bodies like beta-hydroxybutyrate (BHBA) (17, 18), however, these parameters were not part of our diagnostic assessment. Additionally, nearly half of the affected heifers exhibited insufficient neutrophil concentrations, suggesting a compromised immune response and increased susceptibility to infections or inflammatory conditions, potentially due to stress-induced immunosuppression or activation of systemic metabolic inflammation (19). In this study, all heifers with premature lactation exhibited serum cortisol concentrations several times higher than the normal range (4.61–10.29 mmol/L) reported by Di Meo et al. (5), indicating they were under significant stress. This stress could potentially be attributed to factors such as long-distance intercontinental transport, changes in photoperiod during transit (20), or sudden alterations in diet and management practices (21). Notably, altered photoperiods have been shown to affect milk fat percentage and other production traits in dairy cattle, while its potential mechanism on Jersey cows during transport and acclimatization remains unclear (22).

The therapeutic interventions implemented in this case study were effective, demonstrating the importance of targeted management strategies in addressing premature lactation and udder edema in imported heifers. Switching off artificial lights in the barns significantly reduced photosensitivity-related stress, likely associated with mycotoxin exposure (23). The dietary adjustments involving substantial reduction of energy and protein intake likely induced transient metabolic stress but proved to be effective in suppressing milk synthesis and reversing premature udder development. This nutritional strategy replicated the fundamental physiological principle applied during conventional dry-off protocols, where deliberate reduction of dietary nutrient density serves to terminate lactational activity at the end of a normal production cycle. For those showing clinical signs of mastitis, timely interventions, including intramammary antibiotic therapy, anti-inflammatory medications, and regular milking to clear udder congestion, were administered. Clinical mastitis in dairy cows requires prompt intervention to minimize long-term udder damage and maintain productivity. Early management and effective treatment protocols not only resolve acute clinical signs but may also prevent lasting detrimental effects on udder morphology in future lactations by preserving quarter functionality and supporting udder health recovery (24). Studies have shown a strong association between periparturient udder edema and impaired udder conformation, contributing to reduced longevity and lifetime milk yield (25). Additionally, poor udder conformation increases mastitis susceptibility, further compromising production efficiency and farm profitability (26). Thus, implementing timely therapeutic strategies is essential for optimizing dairy herd performance and economic sustainability.

To the best of our knowledge, this is the first documented case report of premature lactation in Jersey heifers following intercontinental air transport. This case was challenging due to the multifactorial nature of the condition, involving transport stress, hormonal imbalances, and feed contamination. Despite these complexities, targeted interventions, including environmental adjustments, dietary changes, and supportive care, could effectively mitigate risks and promote recovery. Meanwhile, it is essential to share this case study with those planning to import pregnant heifers via long-distance transportation. Developing a comprehensive management plan prior to arrival and implementing early interventions for heifers showing clinical signs of premature lactation can attenuate consequences on animal health and productive performance.

## Data Availability

The original contributions presented in the study are included in the article/supplementary material, further inquiries can be directed to the corresponding authors.
